# Epigenetic control of antigen presentation failure in osteosarcoma: from single-cell chromatin maps to therapeutic strategies

**DOI:** 10.3389/fimmu.2025.1728091

**Published:** 2025-11-26

**Authors:** Yan He, Heng Wu

**Affiliations:** Department of Spinal Surgery, The Affiliated Sport Hospital of Chengdu Sport University, Chengdu, China

**Keywords:** osteosarcoma, antigen presentation, DNA methylation, immune evasion, single-cell ATAC-seq, interferon signaling

## Abstract

Osteosarcoma arises within heterogeneous tumor–immune ecosystems in which impaired antigen visibility—shaped by chromatin programs—limits immune surveillance and blunts responses to immunotherapy. Beyond structural defects in the antigen-processing pathway, Polycomb-mediated repression, DNA hypermethylation, and state-specific enhancer closure converge on the HLA class I/NLRC5/interferon axis to diminish peptide display. These constraints are context dependent, varying across malignant clones, differentiation states, and myeloid and T-cell niches. Traditional bulk assays obscure this complexity; single-cell ATAC-seq, integrated with single-cell and spatial transcriptomics, now resolves promoter–enhancer accessibility at HLA, NLRC5, and antigen-processing genes, distinguishes reversible repression from fixed lesions, and links microenvironmental stress to interferon competence. Translationally, epigenetic reprogramming—targeting Polycomb repressive complex 2 (PRC2), DNA methyltransferases (DNMTs), and complementary regulators (for example, LSD1, BET, CDK4/6, YAP/TEAD)—offers biomarker-guided avenues to restore antigen presentation, provided ecosystem-aware pharmacodynamic readouts track chromatin opening and antigen-presentation recovery across compartments. Despite encouraging preclinical evidence, efficacy will depend on clone selection, scheduling that preserves interferon signaling, and rational combinations with innate agonists and checkpoint blockade. This mini-review synthesizes epigenetic mechanisms of antigen-presentation failure in osteosarcoma and outlines how single-cell chromatin profiling can guide strategies to reinstate tumor antigen visibility.

## 1 Introduction

Osteosarcoma is an aggressive primary bone malignancy marked by extensive genomic disruption and profound heterogeneity across malignant, stromal and immune compartments. Recent multi-omics analyses have delineated immune-activated and immune-suppressed osteosarcoma subtypes, underscoring that antitumor immunity varies widely across patients and lesions and that impaired tumor antigen visibility is a recurrent barrier to immune surveillance (1–3). In metastatic and relapsed disease, HLA class I expression and T-cell infiltration correlate, suggesting that defects in antigen presentation shape immune contexture and may limit responsiveness to immunotherapy (4, 5). These observations place the machinery governing peptide processing and HLA class I display at the center of osteosarcoma immune escape.

Antigen presentation failure in cancer arises from both structural lesions in the antigen-processing pathway and reversible transcriptional repression. Beyond mutations or losses in components such as B2M or TAP, tumors co-opt epigenetic programs to silence the MHC class I axis (6–8). Polycomb repressive complex 2 (PRC2) and its catalytic subunit EZH2 can directly suppress genes encoding MHC class I and antigen-processing machinery (APM), thereby diminishing CD8^+^ T-cell recognition; genetic or pharmacologic interference with this pathway restores antigen display and enhances T-cell–mediated cytotoxicity in preclinical models (9–11). In parallel, the MHC class I transactivator NLRC5 is a master regulator of this program; diminished NLRC5—via genetic or epigenetic mechanisms—is associated with reduced HLA class I expression across human malignancies (12–15). These studies establish a mechanistic link between chromatin state and tumor immunogenicity and provide a rationale to interrogate epigenetic control of the antigen-presentation axis in osteosarcoma.

Osteosarcoma is characterized by widespread epigenomic dysregulation, including oncohistone alterations and aberrant DNA methylation, which influence lineage programs and microenvironmental interactions. Although the specific consequences for the antigen-presentation network in osteosarcoma are incompletely defined, emerging evidence indicates that epigenetic therapies can reprogram tumor cells toward greater immune visibility by increasing expression of HLA class I components and interferon-stimulated genes (16, 17). In osteosarcoma models, DNA demethylation reshapes transcriptional programs, supporting the plausibility that chromatin-level interventions could intersect with antigen processing (18–20). More broadly across cancers, inhibition of DNA methyltransferases (DNMTs) can induce viral mimicry with upregulation of interferon-stimulated genes (ISGs) and antigen-presentation machinery, providing a conceptual framework to test similar strategies in osteosarcoma.

Single-cell assay for transposase-accessible chromatin sequencing (scATAC-seq) now enables direct, cell-resolved measurement of chromatin accessibility at promoters and enhancers of HLA genes, NLRC5 and interferon-pathway effectors within complex tumor ecosystems. Applied alongside single-cell and spatial transcriptomics, scATAC-seq can map how malignant clones, osteoblastic differentiation states and myeloid or lymphoid niches converge to establish epigenetic bottlenecks to antigen presentation (21–23). By capturing both inter- and intra-tumoral heterogeneity in regulatory element usage, scATAC-seq provides a mechanistic lens to distinguish fixed lesions from reversible repression and to identify candidate nodes—such as PRC2 occupancy or distal enhancers of NLRC5—for therapeutic modulation (24, 25). This mini-review synthesizes current knowledge on epigenetic control of antigen presentation failure in osteosarcoma and outlines how single-cell chromatin profiling can guide strategies to restore tumor antigen visibility.

## 2 Osteosarcoma chromatin constraints on antigen presentation

Osteosarcoma exhibits pervasive epigenomic remodeling that can directly constrain the MHC class I axis. In many solid tumors, Polycomb repression contributes to immunoediting by depositing H3K27me3 across promoters and enhancers of the MHC class I transactivator NLRC5 and antigen-processing machinery, thereby reducing HLA class I display; pharmacologic inhibition of PRC2 components restores MHC class I expression in preclinical systems, supporting a causal role for chromatin state in antigen visibility (26–28). Within this framework, NLRC5 functions as a lineage-agnostic master regulator of MHC class I genes and several processing components (for example, TAP1, PSMB8/9), and its diminished expression—by genetic or epigenetic mechanisms—links inaccessible chromatin to ineffective peptide presentation (29, 30). In osteosarcoma specifically, EZH2 activity is frequently elevated and mechanistically connected to undifferentiated programs; although the immunologic consequences have not been fully mapped in patient material, these observations align with a model in which PRC2 activity imposes a reversible ceiling on antigen presentation capacity in subsets of tumors.

DNA methylation patterns provide a second axis of constraint. Osteosarcoma harbors reproducible methylation signatures that stratify risk and delineate disease subtypes, and epigenetic reactivation experiments in osteosarcoma cells demonstrate that demethylation can derepress immune-related transcripts (31–33). Across cancers, inhibition of DNA methyltransferases—alone or combined with histone deacetylase inhibition—can elicit a viral-mimicry response with double-stranded RNA accumulation, type I/II interferon signaling, and upregulation of the antigen-presentation machinery, providing a conceptual route to restore tumor visibility; these effects, established in multiple models, motivate testing in osteosarcoma contexts with appropriate pharmacodynamic readouts (34, 35). At the tissue level, osteosarcoma specimens show heterogeneous but interpretable relationships between HLA class I expression and lymphocyte infiltration, consistent with the notion that chromatin-encoded suppression of antigen presentation shapes immune contexture.

Single-cell chromatin accessibility profiling offers a practical means to resolve these constraints within complex tumor ecosystems. scATAC-seq can quantify accessibility at proximal promoters and distal enhancers for HLA genes, NLRC5, and interferon-response effectors, and can attribute repressive signatures (for example, H3K27me3-associated inaccessibility or loss of IRF/RFX motif accessibility) to specific malignant clones and differentiation states (36–38). In parallel, joint single-cell chromatin and transcriptome profiling helps distinguish fixed structural lesions from reversible repression, enabling prioritization of epigenetic targets most likely to restore antigen processing (39–41). An operational summary of candidate chromatin constraints in osteosarcoma and their expected single-cell readouts is provided in **Table 1**, which is intended to guide experimental design and interpretation in forthcoming studies. Osteosarcoma is poised for an integrated epigenetic–immunologic framework in which PRC2 activity, DNA methylation, and state-specific enhancer usage converge to limit peptide display. Cell-resolved chromatin maps can identify patients and clones with reversible repression, nominate precise regulatory elements for modulation, and provide quantitative pharmacodynamic endpoints for trials testing PRC2 or DNA-methylation–directed strategies aimed at restoring tumor antigen visibility.

**Table 1:** Candidate chromatin constraints on antigen presentation in osteosarcoma and expected single-cell readouts.

Constraint	Primary chromatin feature	Expected scATAC-seq signature	Predicted transcriptional consequence	Candidate reversible interventions
PRC2/EZH2-mediated repression at NLRC5/HLA and APM loci	H3K27me3-dominated promoters/enhancers	Reduced accessibility at NLRC5, HLA-A/B/C, TAP1, PSMB8/9 regulatory elements; depleted RFX/IRF motif activity	Lower NLRC5 and APM transcripts; diminished HLA class I surface expression	EZH2/EED inhibition; interferon-γ priming
DNA hypermethylation of APM genes and interferon-response enhancers	CpG promoter/enhancer methylation	Reduced peak intensity at CpG-dense promoters; loss of STAT/IRF-linked enhancers	Attenuated interferon-stimulated gene programs and APM expression	DNMT inhibition; dual DNMT/HDAC inhibition
Loss of distal enhancer activity for antigen-presentation circuitry	Silenced distal enhancers	Closure of distal peaks harboring RFX/STAT motifs	Incomplete induction of HLA axis under inflammatory cues	Pathway-targeted chromatin modulation; cytokine stimulation
Malignant differentiation state with skewed TF usage	Lineage-biased motif landscape	Elevated RUNX2/OSX motifs with concurrent loss of RFX/IRF accessibility	Context-dependent reduction in HLA/APM transcription	Differentiation-oriented therapies with immune monitoring

## 3 Single-cell ATAC-seq profiles of the osteosarcoma tumor–immune ecosystem

**Figure 1:**
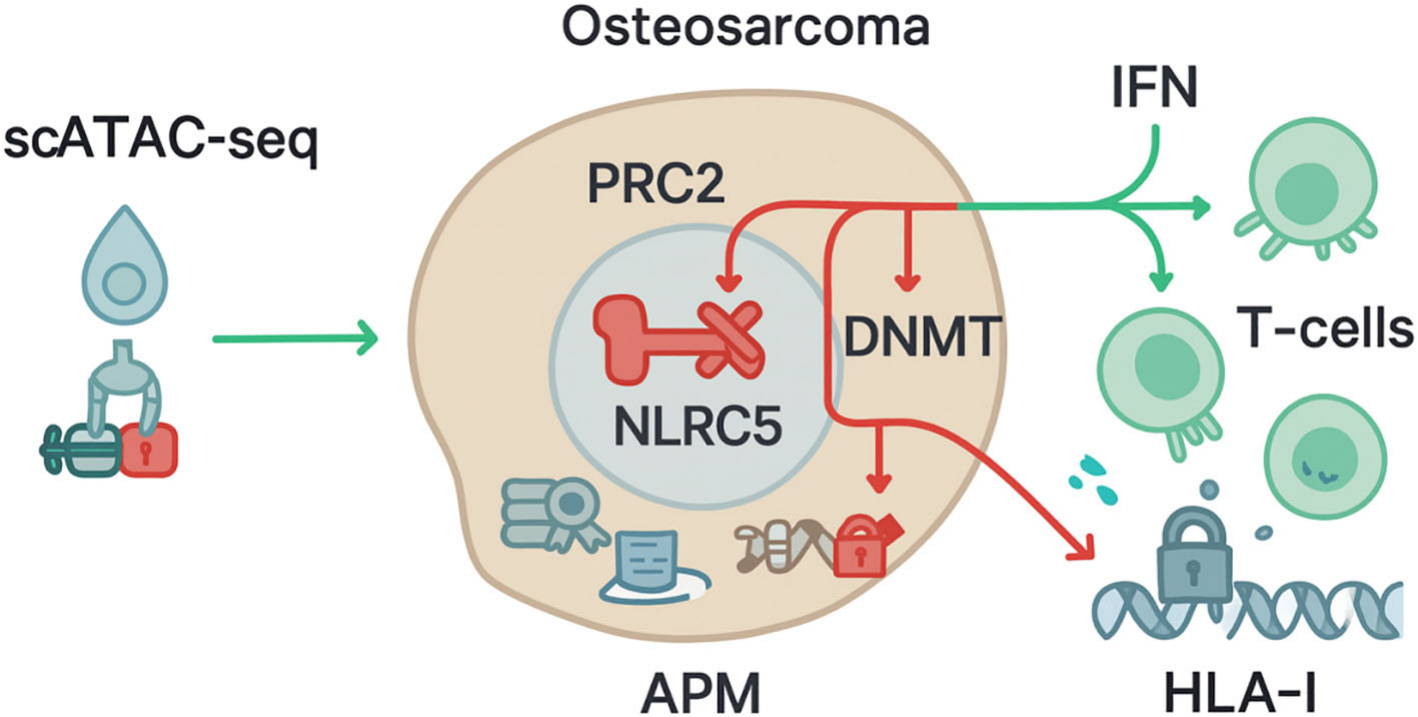
scATAC-seq reveals epigenetic suppression of the NLRC5–HLA-I/APM axis limiting T-Cell recognition in osteosarcoma.

Single-cell chromatin accessibility profiling resolves how malignant, stromal, and immune compartments in osteosarcoma jointly encode constraints on antigen visibility. In malignant cells, scATAC-seq delineates clone-specific enhancer–promoter usage across HLA class I genes, NLRC5, and antigen-processing machinery, while simultaneously exposing differentiation-state dependencies that couple osteoblastic lineage programs to immunogenicity (42, 43). As shown in **Figure 1**, accessibility losses at RFX/IRF/STAT-bearing regulatory elements near HLA-A/B/C, TAP1, and PSMB8/9, together with diminished NLRC5 enhancer activity, are recurrent features in immune-cold tumor regions and correspond to reduced interferon-response competence, consistent with prior single-cell and bulk chromatin studies in solid tumors (44, 45). Copy-number–aware scATAC-seq and peak–gene linkage analyses distinguish irreversible structural lesions from reversible repression, allowing inference of candidate nodes—such as PRC2-dominated promoters or silenced distal enhancers of NLRC5—whose modulation restores antigen presentation in preclinical systems (46–48). Integration with matched single-cell RNA-seq further validates that local closure at these elements aligns with attenuated HLA and antigen-processing transcripts rather than global transcriptional collapse (49, 50), supporting a specific epigenetic mechanism rather than nonspecific stress effects.

The immune landscape extracted from scATAC-seq adds mechanistic resolution to T-cell dysfunction and myeloid-driven suppression in osteosarcoma. Exhausted CD8^+^ T cells exhibit increased TOX and NFAT motif accessibility with reduced AP-1/IRF co-accessibility at interferon-inducible enhancers proximal to antigen-presentation targets, mirroring transcriptional states that associate with impaired cytotoxic function and poor tumor control in sarcomas (51–53). Regulatory T cells show strong FOXP3 and NF-κB motif accessibility and co-accessible chromatin at immunosuppressive cytokine loci, providing a structural correlate for sustained checkpoint pathway activity (54, 55). On the myeloid axis, scATAC-seq resolves macrophage continua from inflammatory to immunoregulatory states; in tumor-proximal macrophages, decreased IRF/STAT motif activity at antigen-presentation modules coexists with enhanced accessibility at enhancers controlling ARG1, IL10, and TGFB1, consistent with impaired cross-priming and active T-cell suppression (56–58). Dendritic cell subsets with accessible BATF3/IRF8 programs are typically sparse in immune-cold niches, and where present, exhibit attenuated accessibility at CCR7 and costimulatory gene enhancers, suggesting defective maturation and trafficking.

Chromatin-level cell–cell interactions inferred from co-accessibility and ligand–receptor–anchored enhancer activity connect ecosystem structure to antigen-presentation failure. Osteosarcoma cells with high EZH2-linked repression programs co-localize with myeloid niches that display reduced IRF accessibility, indicating convergent dampening of interferon pathways across compartments (59–61). Spatially informed analyses align low HLA enhancer accessibility with hypoxic regions marked by HIF-associated motif gains, raising the possibility that microenvironmental stress reprograms chromatin to limit peptide display independently of fixed genetic lesions (62–64). These patterns are reproduced across metastatic deposits, where clonal enhancer switching and continued loss of NLRC5 regulatory accessibility track with immune exclusion and attenuated CD8^+^ infiltration.

Robust inference requires rigorous quality control and analytical standardization. Nucleosomal signal, transcription-start-site enrichment, doublet removal, and batch correction are essential to prevent artifactual loss of accessibility at compacted loci (65, 66). Peak calling tailored to sparse single-cell data, chromVAR-based motif deviation scoring, and peak–gene linkage within frameworks such as ArchR or Cicero provide reproducible quantification of regulatory programs that govern antigen presentation (67–69). Allele-specific accessibility at HLA loci can be incorporated to separate haplotype loss from epigenetic repression, and perturbation-coupled scATAC-seq offers direct pharmacodynamic readouts for epigenetic therapies that seek to restore antigen visibility (70–72). These single-cell chromatin maps define an osteosarcoma tumor–immune ecosystem in which malignant enhancer architecture, myeloid-driven interferon desensitization, and T-cell exhaustion converge on a shared endpoint of reduced HLA class I display, thereby establishing measurable, targetable epigenetic bottlenecks to antigen presentation.

## 4 Epigenetic strategies to restore antigen visibility in osteosarcoma

Epigenetic interventions aimed at reversing chromatin constraints on the MHC class I axis provide a rational path to increase tumor antigen visibility in osteosarcoma. Inhibition of Polycomb repressive complex 2 can relieve promoter–enhancer repression across NLRC5 and antigen-processing loci; across cancer models, EZH2 blockade restores MHC class I programs and improves immune recognition, supporting this strategy where PRC2 activity is heightened (73–75). Mechanistically, EZH2 inhibition also raises stimulator of interferon genes (STING) pathway competence, offering a tractable avenue for combination with cyclic dinucleotide agonists to amplify interferon-driven antigen presentation (76, 77). These concepts, supported predominantly by preclinical data and not yet validated in osteosarcoma-specific trials, align with single-cell chromatin evidence of reversible repression at HLA/NLRC5 regulatory elements and motivate biomarker-guided exploration in early-phase studies.

DNA methylation is a second, actionable axis: inhibition of DNA methyltransferases (DNMTs) and histone deacetylases (HDACs) induces viral mimicry (double-stranded RNA; type I/III interferon), elevating antigen presentation and immunogenicity (78–80). Candidates show scATAC-seq evidence of CpG-dense, closed promoters and interferon-responsive enhancers near NLRC5, TAP1, PSMB8/9 (81, 82). Scheduling with cytokine priming or innate agonists maximizes reprogramming while limiting cytotoxicity. Orthogonal tests: lysine-specific demethylase 1 (LSD1) blockade boosts MHC-I, dendritic chemokines, and checkpoint efficacy in small-cell/myeloid and osteoblastic–RFX/IRF-low states (83, 84); bromodomain and extra-terminal domain (BET) blockade may raise MHC-I yet dampen dendritic activation, requiring compartment-resolved pharmacodynamics (85, 86). scATAC-seq–anchored trial designs should therefore incorporate myeloid and T-cell accessibility metrics to anticipate ecosystem-level effects.

Cell-state and signaling–directed epigenetic combinations can further restore peptide display. CDK4/6 inhibitors promote endogenous retroelement expression and interferon signaling, increase antigen-presentation gene expression, and reduce regulatory T-cell proliferation; pairing CDK4/6 blockade with PRC2 or DNMT-directed agents could align tumor-intrinsic antigen restoration with favorable immune composition (87–89). Hippo pathway modulation provides an additional axis: YAP/TEAD inhibition upregulates NLRC5 and antigen-processing genes, offering a route to reopen distal enhancers mapped by scATAC-seq (90, 91). For osteosarcoma, these strategies should be advanced with prospective selection of clones exhibiting PRC2-dominated inaccessibility or methylation-linked enhancer closure (**Table 2**), and with quantitative single-cell pharmacodynamic endpoints.

**Table 2:** Epigenetic strategies to restore antigen visibility in osteosarcoma.

Strategy	Primary target/process	Expected scATAC-seq hallmarks at baseline	Pharmacodynamic success indicators	Logical combinations	Key caveats
PRC2 inhibition (EZH2/EED)	Relief of H3K27me3 repression at NLRC5/HLA/APM	Closed promoters/enhancers with depleted RFX/IRF/STAT motifs at HLA-A/B/C, NLRC5, TAP1, PSMB8/9	Increased accessibility and motif deviation at NLRC5/HLA enhancers; induction of APM transcripts; increased HLA-I surface	STING agonists; IFN-γ priming; PD-1/PD-L1 blockade	Dose/schedule to avoid myelosuppression; monitor off-tumor interferon toxicity
DNMT (± HDAC) inhibition	Demethylation; viral-mimicry interferon signaling	CpG-dense promoter closure and inaccessible ISG enhancers	Reopened CpG-rich promoters; ISG/chemokine induction; restored HLA-I	Pattern-recognition receptor (PRR)/STING agonists; CDK4/6 inhibitors	Transient lymphopenia; need for interferon-competent clones
LSD1 inhibition	Reprogramming of lineage/immune genes enhancing MHC-I	Lineage-skewed motifs with low RFX/IRF accessibility	Increased accessibility at APM enhancers; higher MHC-I and DC-recruiting chemokines	PD-1/PD-L1 blockade; differentiation therapy	Context-dependent effects on tumor differentiation
BET inhibition	Relief of super-enhancer constraints; MHC-I upregulation	Silenced distal enhancers near APM circuitry	Restored distal enhancer accessibility; antigen-presentation gene induction	Checkpoint blockade; innate agonists	Potential dendritic-cell functional suppression—track myeloid accessibility
CDK4/6 inhibition	dsRNA/IFN induction; Treg control	Cell-cycle–linked enhancer use; partial closure at ISG/APM	APM/ISG upregulation; increased antigen-presentation transcripts; reduced Treg signatures	DNMT/PRC2 inhibitors; PD-1/PD-L1 blockade	Schedule to separate cytostatic and immunogenic windows
YAP/TEAD inhibition	NLRC5/APP enhancer activation	Low NLRC5 enhancer accessibility	NLRC5 and APM enhancer opening with RFX/STAT motif gain	With PRC2 or DNMT inhibitors	Pathway redundancy; monitor skeletal lineage effects

## 5 Conclusions and outlook

Epigenetic interventions that reopen the MHC class I axis offer a tractable route to increase tumor antigen visibility in osteosarcoma while accommodating its inter- and intra-tumoral heterogeneity. Inhibiting Polycomb repressive complex 2 (PRC2) can derepress promoters and distal enhancers across NLRC5 and antigen-processing genes, restoring HLA class I and sensitizing tumors to T-cell attack in diverse preclinical systems (92, 93). Mechanistic work shows that PRC2/EZH2 maintains a conserved silencing program over the antigen-presentation pathway, and pharmacologic EZH2/EED inhibition reverses this constraint and augments response to immunotherapy *in vivo*. Taken together, these data support consideration of PRC2-directed clinical studies, particularly when single-cell maps indicate H3K27me3-dominated inaccessibility at NLRC5/HLA regulatory elements, while recognizing that osteosarcoma-specific trial data are still limited (94, 95). Moreover, EZH2 blockade can potentiate innate-sensing pathways, including STING, providing a rationale for combinations with cyclic dinucleotide agonists to amplify interferon-driven upregulation of antigen-presentation machinery.

DNA methyltransferase (DNMT) inhibition represents a complementary lever that induces “viral mimicry,” with endogenous double-stranded RNA accumulation, type I/III interferon signaling and coordinated induction of HLA and antigen-processing programs (96, 97). These effects have been demonstrated across epithelial models and provide a framework for testing demethylating agents in osteosarcoma with pharmacodynamic readouts anchored in interferon competence and chromatin opening at CpG-dense promoters and interferon-responsive enhancers (98–100). Pairing DNMT inhibition with CDK4/6 inhibitors may couple tumor-intrinsic restoration of antigen presentation to favorable immune remodeling, as CDK4/6 blockade increases tumor cell antigen-presentation gene expression and reduces regulatory T-cell proliferation, enhancing cytotoxic responses. Nonetheless, support for these epigenetic–immunologic strategies is currently derived mainly from preclinical models, particularly for osteosarcoma.

Targeting additional chromatin regulators may widen the therapeutic aperture. LSD1 inhibition can upregulate MHC-I and dendritic-cell–recruiting chemokines, reprogramming poorly immunogenic states and improving responsiveness to checkpoint blockade; these effects argue for state-aware deployment when lineage motifs coincide with low RFX/IRF accessibility at antigen-presentation enhancers (101, 102). BET bromodomain inhibition has also been shown to relieve repressive control over antigen-presentation circuitry and to engage antitumor immunity (103, 104), nominating combinations with PD-1/PD-L1 inhibitors while calling for compartment-resolved pharmacodynamics to track possible myeloid effects.

Osteosarcoma-specific epigenomic features underscore the feasibility of this agenda. Risk-stratifying methylation subtypes and decitabine-responsive programs highlight a disease in which DNA methylation intersects with tumor–stromal interactions and may be exploited to reinstate immune visibility (105–107). These signatures, together with integrative analyses linking methylation to gene expression in osteosarcoma, support biomarker-guided selection of demethylating or Polycomb-targeted strategies.

Looking to the Future, single-cell ATAC-seq should be embedded prospectively to select patients with reversible repression at NLRC5/HLA and antigen-processing loci, to nominate distal enhancers for modulation, and to deliver quantitative pharmacodynamic endpoints that distinguish true antigen-restorative effects from nonspecific stress responses. Beyond PRC2 and DNMT axes, Hippo pathway modulation is an emerging avenue: YAP/TEAD inhibition relieves repression of NLRC5 and increases MHC-I antigen-processing gene expression, offering a differentiation-aware route to reopen distal enhancers. Trials should incorporate matched single-cell chromatin and transcriptomic profiling across malignant, myeloid and T-cell compartments, with allele-specific interrogation at HLA loci and standardized interferon-response metrics. The strategic goal is to convert antigen-poor osteosarcoma clones into interferon-competent, HLA-replete states that admit durable T-cell control, using epigenetic agents as enabling therapies in rational combinations with innate agonists and checkpoint blockade.
